# A study of clinical correlates and socio-demographic profile in conversion disorder

**DOI:** 10.4103/0019-5545.37323

**Published:** 2007

**Authors:** Kamala Deka, Pranit K. Chaudhury, Kavery Bora, Pranab Kalita

**Affiliations:** Department of Psychiatry, Assam Medical College and Hospital, Dibrugarh, Assam, India

**Keywords:** Conversion, dissociation, precipitating factor

## Abstract

**Aim:**

To study the clinical presentations and relationship of socio-demographic variables with conversion disorder.

**Methods:**

Forty patients admitted to the department of psychiatry, Assam Medical College and Hospital, Dibrugarh, during November 2004 to August 2005 who fulfilled the inclusion criteria of the study were evaluated for socio-demographic variables and clinical presentations on a semi-structured pro forma.

**Results:**

Conversion disorder is more common in young adults (57.5%), females (92.5%) and among students belonging to nuclear family of lower socioeconomic status. A majority of the patients had an obvious precipitating factor, of which family-related (40%) and school-related (30%) problems accounted for the major types. Motor symptoms were the predominant presentation (87.5%) with pseudo seizure being the commonest.

Conversion disorder is characterized by the presence of deficits affecting the voluntary motor/sensory functions without any organic basis, while excluding the symptoms fully explainable by a general medical condition, substance abuse or culturally sanctioned behavior. The presenting symptoms are unintentional and may mimic a neurological disorder. Hysteria patients constitute a major proportion of psychiatric patient population in developing countries.[1–3] Some Indian studies have focused on the clinical characteristics in conversion disorder.[4,5] They have emphasized on the role of stressors in conversion disorder. “Role model”; has been reported in conversion disorder in some earlier studies (Sridhar and Sudharkar, 1997).[13] A role is an automatic learned, goal-directed pattern or sequence of acts developed under the influence of significant people in a growing child's environment. Patients with conversion disorder may unconsciously model their symptoms on those of someone important to them. In India, high occurrence of conversion disorder has been reported in young adults, from poor low-income, joint families, and significantly higher in females.[6] Also, higher prevalence has been seen in illiterates, married housewives being the largest group.[7] But less is known from this region about the clinical presentations and socio-demographic variables in conversion disorder.

This study is an effort to know the various types of clinical presentations and the related socio-demographic variables in conversion disorder in this part of upper Assam.

## MATERIALS AND METHODS

Forty new cases (every second) admitted to the Dept. of Psychiatry, Assam Medical College and Hospital, Dibrugarh, during November 2004 to August 2005 who fulfilled the inclusion criteria of the study were enrolled for the study.

### Inclusion criteria

Subjects of both sexes of age 6 years and above and fulfilling diagnostic criteria of dissociative (conversion) disorder according to ICD-10 were included.[8]

### Exclusion criteria

Subjects having known history of organic disorder, including epilepsy and comorbid other psychiatric illness, e.g., anxiety disorder, depressive disorder, etc., were excluded.

### Tools used

1. The ICD-10 classification of mental and behavioral disorders[8]
2. A semi-structured pro forma to record socio-demographic details, including age, sex, education, occupation, domicile, marital status, family type and socioeconomic status; in addition to birth order, role model, clinical presentations and precipitating factor for developing dissociative (conversion) disorder.

### Procedure of study

All the study subjects were thoroughly evaluated on the basis of history and mental status examination, and the diagnosis was confirmed by a senior psychiatrist.

Then, the consent was taken from every patient before enrolling into the study. All the patients and their attendants were then evaluated to elicit necessary information required in our semi-structured pro forma.

### Analysis of data

Data were analyzed by using Karl Pearson's correlation coefficient (Chi-square test).

## RESULTS

The socio-demographic characteristics of the subjects are summarized in Table 1. A majority of the subjects were female (92.5%), unmarried (72.5%) and were in the age range of group 18-29 years (57.5%), followed by 6-17 years age group (30%). Half of the study subjects were students (50%), followed by housewives (20%). Most of the subjects were literates (80%). The difference between illiterates and literates was statistically significant (X^2^ = 14.400, df = 1, *P* = 0.000). A majority of the subjects had a rural background (42.5%), followed by those from tea gardens (30%), and were from a nuclear family (82.5%). Most of our study subjects had low socioeconomic status (75%).

**Table 1:** Socio-demographic profile of the subjects (n = 40)

Variable	Variants	Conversion disorder		
		
		n (%)	X^2^	*P*
Sex	Female	37 (92.5)	28.90	0.000
	Male	3 (7.5)	df = 1	S
Age (in year)	6-17	12 (30)	28.60	0.000
	18-29	23 (57.5)	df = 3	S
	30-41	3 (7.5)		
	42-53	2 (5)		
Marital status	Single	29 (72.5)	8.100	0.004
	Married	11 (27.5)	df = 1	S
Occupation	Unemployed	6 (15)		
	Employed	6 (15)	13.60	0.004
	Housewife	8 (20)	df = 3	S
	Student	20 (50)		
Literary level	Illiterate	8 20)	14.400	0.000
	Literate	32 (80)	df = 1	S
Locality	Rural	17 (42.5)		
	Urban	11 (27.5)	1.550	0.461
	Tea garden	12 (30)	df = 2	NS
Family type	Nuclear	33 (82.5)	16.90	0.000
	Joint	7 (17.5)	df = 1	S
Socio economic status	Lower	30 (75)	10.0	
	Middle	10 (25)	df = 1	0.002
	Upper	0	0	S

NS = Nonsignificant, S = Significant, df = Degree of freedom

Role models were present in 52.5% of the subjects. We had checked for the birth order of the subjects and found that most of the subjects were the third (27.5%) or second (25%) or the single child of the family (25%).

Motor symptoms were the most common type of clinical presentation (87.5%). Amongst the motor symptoms, pseudo seizure was the commonest presentation (71.4%). Other motor symptoms included paresis (17.1%), aphonia/dysphonia (20%), hyperventilation (17.1%), dizziness (14.3%), limb paralysis (5.7%) and astasia abasia (5.7%).

No subjects presented with isolated sensory symptoms.

As many as 7.5% of the subjects presented with “other symptoms,” while only 5% subjects presented with “dissociative symptoms” (dissociative stupor).

“Other symptoms” category included two cases of mixed dissociative [conversion] disorders and one case of Ganser's syndrome.

Subjects from all the three localities (rural, urban and tea garden) predominantly presented with motor symptoms. While dissociative symptoms were presented only by the subjects from rural and tea garden community, “other symptoms” were mainly presented by subjects from urban area.

We have also evaluated whether the subjects had any obvious precipitating factor prior to onset of illness, and we found that 40% subjects had family-related problems, 30% had school-related problems and the rest 30% had “love affair”-related problems. Family-related and “love affair”-related precipitating factors have positive association with increasing age, whereas study- /school-related factors have negative association with age.

## DISCUSSION

In this study occurrence of conversion disorders was found to be higher in females (92.5%) than in males (7.5%), and a majority of our patients were young adults in the 18-29 years age group (57.5%), followed by those in the 6-17 years age group (32.5%). This corresponds with the findings by Vyas *et al*,[6] Bagadia *et al*[9] and Choudhury *et al*.[10] Moreover, these findings obviously support already established findings of prevalence of conversion disorder. Majority of our subjects were literate (80%). Though there were literates, they had not reached a very high educational level; and most of them had completed only 10 or less than 10 years of formal education. The predominant study population was of students (50%) and hence they were unmarried (72.5%). This is in contrast with the findings by Jain and Verma *et al.*[11] and Choudhury *et al.*,[10] who found housewives and married to be the predominant group. As many as 42.5% of the subjects belonged to the rural community and 30% to the tea garden community. Therefore, it can be presumed that a majority of the subjects had a rural background as the tea gardens are usually located in the rural areas. As many as 82.5% of the study population were from nuclear families, which could possibly be due to life-style pattern changing to a modernized one. This is not in keeping with the findings by Vyas *et al*.[6] Most of the subjects had lower socioeconomic status (75%). Role models were present in 52.5% of the cases.

Motor symptoms were the commonest presentation (87.5%), irrespective of the community status or literacy level, of which pseudo seizure (71.4%) was the commonest. This is in contrast to the findings by Roelofs *et al.*,[12] who found paresis/paralysis to be the commonest. Dissociative symptoms were presented only by the illiterates of rural or tea garden community. While 37.5% of illiterate patients had not taken any treatment before being brought to the psychiatry department, 62.5% of literate patients had opted for prior medical treatment, suggesting an association between literacy level and medical treatment acceptance.

While study- /school-related factors were the major precipitating factors in children and adolescents (66.6%), it was the family-related factors (100%) in the 30-41 years age group. “Love affair”-related factors showed a significant rise from the childhood and adolescents group (8.4%) to the young adult group (26.1%).

### Limitations of the study

The sample size was small. As this was a cross-sectional study, the pattern of symptomatology in subsequent recurrence could not be studied thereof.

However, the study could have evaluated the cultural influence on the presentation of symptoms in the three different localities. Further research is needed with bigger sample to validate and replicate our findings.
